# Superlubricity of Graphite Sliding against Graphene Nanoflake under Ultrahigh Contact Pressure

**DOI:** 10.1002/advs.201800810

**Published:** 2018-08-29

**Authors:** Jinjin Li, Jianfeng Li, Jianbin Luo

**Affiliations:** ^1^ State Key Laboratory of Tribology Tsinghua University Beijing 100084 China

**Keywords:** graphene, graphite, nanotribology, superlubricity, ultrahigh pressures

## Abstract

Superlubricity of graphite sliding against graphene can be easily attained at the nanoscale when it forms the incommensurate contact under a low contact pressure. However, the achievement of superlubricity under an ultrahigh contact pressure (>1 GPa), which has more applications in the lubrication of micromachine and nanomachine, remains unclear. Here, this problem is addressed and the robust superlubricity of graphite is obtained under ultrahigh contact pressures of up to 2.52 GPa, by the formation of transferred graphene nanoflakes on a silicon tip. The friction coefficient becomes as low as 0.0003, a state that is attributed to the extremely low shear strength of the graphene/graphite interface in the incommensurate contact. When the pressure exceeds some threshold, the superlubricity state collapses suddenly with the friction coefficient increasing ≈10 times. The failure of superlubricity originates from the delamination of the topmost graphene layers on graphite under ultrahigh contact pressures, which requires the tip to provide additional exfoliation energies during the sliding process. The results demonstrate that the superlubricity of graphite sliding against graphene can exist stably under ultrahigh contact pressure, which would appear to accelerate its application in nanoscale lubrication.

## 1 Introduction

In the design of high‐performance micro‐electromechanical systems or nano‐electromechanical systems (MEMS/NEMS), extremely low friction and wear are crucial because they directly determine the working efficiency and life of these devices.^1^ Superlubricity, originally describing the phenomenon that the friction between two sliding surfaces vanishes or very nearly vanishes,^2^ is considered one of the most effective methods to achieve extremely low friction.^3^ This state may occur when two crystalline surfaces slide over each other in dry incommensurate contact without wear and deformation, which is also called structural superlubricity.^4^ Owing to the weak van der Waals (vdW) interaction among interlayers, the superlubricity between 2D layered materials, such as graphene, boron nitride, molybdenum disulfide (MoS_2_), and graphite has recently been widely studied.[[qv: 4c,5]] Such materials exhibit promising lubrication properties from atomic‐scales to micro‐scales in specific sliding orientations. For example, the friction nearly vanishes when the nanoscale graphite flake slides on the graphite,^6^ micrometer‐scale graphite island self‐retracts on the graphite,^7^ and MoS_2_ monolayers slide on a MoS_2_ substrate,[[qv: 5c]] because of the formation of structural superlubricity with the incommensurate surface lattices. By contrast, some specific dissimilar friction pairs, like crystalline gold nanoparticles sliding on graphite and graphene nanoribbons sliding on gold, also exhibit superlubricity at the nanoscale.^8^

However, these near‐frictionless sliding systems have only been achieved at extremely low (or even negative) loads or pressures. The loads in some cases only originate from the gravity of the flakes and cohesive force between two sliding surfaces with a contact pressure of less than 1 MPa, while the others originate from the small normal force applied by atomic force microscopy (AFM) tip/probe (Table S1 of the Supporting Information gives a summary of loads/pressures for superlubricity of graphite or graphene). Such low contact pressures limit widespread applications in MEMS/NEMS lubrication, where the contact pressure for sliding parts can reach up to the GPa scale.^9^ Although it is challenging to translate the structural superlubricity into high contact pressure, several recent experimental studies have reported the emergence of superlubricity under high contact pressure. The superlubricity between a silica probe and graphite was achieved at a local contact pressure of up to 700 MPa through the formation of multiple transferred graphene nanoflakes (GNFs),^10^ and the superlow friction was achieved between diamond‐like carbon and graphene through the formation of graphene nanoscrolls on tribometer.^11^

It is expected that the high contact pressure naturally limits superlubricity, because a new energy dissipation channel may be introduced when the contact pressure is high enough to produce notable deformations on the two sliding surfaces.^12^ In the case of such large deformations, we can no longer describe the situation as composed of two rigid bodies, which may exhibit unusual superlubricity behavior. Therefore, in this study, we obtained the robust superlubricity of graphite by the formation of transferred GNFs on an AFM tip, and then investigated the superlow friction behavior, extra energy dissipation channel, and failure mechanism of superlubricity under ultrahigh contact pressures.

## 2 Results

**Figure 1:**
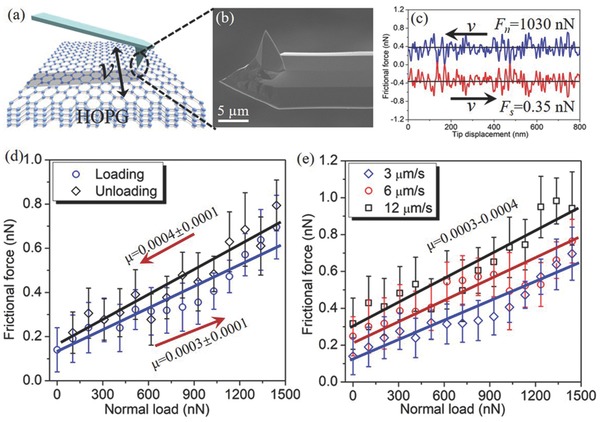
a) Illustration of AFM tip sliding on the HOPG under a constant normal load. b) Field emission scanning electron microscopy (FESEM) image of the AFM tip. c) Typical frictional force loop under a constant load of 1030 nN after the presliding. d) Frictional forces as functions of normal loads between AFM tip and HOPG, measured during loading and unloading process. The full lines are the linear fits to these points, giving friction coefficients of 0.0003 ± 0.0001 (loading) and 0.0004 ± 0.0001 (unloading). The sliding speed was set to 3 µm s^−1^. e) Frictional forces as functions of normal loads between AFM tip and HOPG measured under three different sliding speeds (3, 6, and 12 µm s^−1^). The full lines are the linear fits to these points, giving the friction coefficient range of 0.0003–0.0004.

The highly ordered pyrolytic graphite (HOPG) was freshly cleaved using the adhesive tape to give a clean, atomically flat surface soon before each friction test. The frictional forces were measured by driving the freshly cleaved HOPG sliding against a sharp silicon tip (tip radius is about 7 nm, Figure S1, Supporting Information) under an applied load (**Figure** **1**a,b). Figure 1c shows a typical frictional loop under a constant load of *F*_n_ = 1030 nN after the presliding (to stabilize the frictional force), with the result showing an extremely small frictional force of *F*_s_ = 0.35 nN. Thus, the ratio of *F*_s_ to *F*_n_, equal to friction coefficient approximately, is calculated as 0.0003. More than ten independent silicon tips were performed to measure the frictional forces, all of which exhibited the similarly small friction. Moreover, such small frictional force was maintained as the tip slid on the HOPG for a long distance of up to 10^4^ µm (Figure S2, Supporting Information), demonstrating the robustness of this extremely low friction system.

The frictional forces as functions of normal loads were obtained by increasing the load gradually (loading) and then reducing the load over the same interval (unloading), as shown in Figure 1d. The frictional forces were observed to increase linearly during the loading process and decrease linearly during the unloading process as the load varied from 0–1500 nN. The friction coefficients, defined by the slope of the two linear fits to these points, are extremely low for both loading process (*µ* = 0.0003 ± 0.0001) and unloading process (*µ* = 0.0004 ± 0.0001), and are clearly within the superlubricity regime.[[qv: 3a]] The small difference in friction between the loading and unloading process indicates that there is no remarkable hysteresis behavior (the loading friction is smaller than the unloading friction), unlike an AFM tip sliding on graphene or graphite under low pressure.^13^ Meanwhile, an offset frictional force was observed when the applied load was zero, indicative of the existence of an adhesive force between the tip and HOPG.

The influence of sliding velocity on the superlubricity was analyzed by measuring the frictional force under three different scanning speeds (Figure 1e). The frictional behavior under these different sliding speeds was almost the same; that is all the frictional forces maintained superlow values and increased slowly with increasing load until the load reached 1500 nN. Although there was slight increase in the frictional force at higher velocity for a constant load, the friction coefficients (slope of fitting lines) were all in the range of 0.0003–0.0004, exhibiting the weak dependence of sliding velocity on superlubricity. Meanwhile, the HOPG substrate was rotated with respect to the tip with an angle range of 120° to study the influence of the sliding orientations on the frictional force of graphite. The frictional forces remained almost constant (no peak was observed) with the variation of sliding orientation under a constant load (Figure S3, Supporting Information). It did not exhibit the sliding orientation dependent superlubricity, which is different from previous studies showing that the superlubricity of graphite disappeared at two certain sliding orientations with an angle difference of 60°.^6^, ^7^ Therefore, it is inferred that the friction behavior of graphite under high contact pressures may be different from that under extremely low pressures.

**Figure 2:**
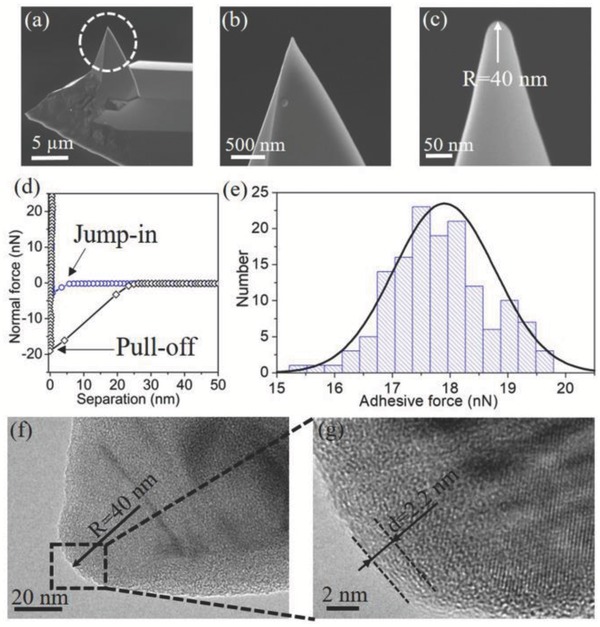
a) FESEM image of AFM tip after the friction test. b,c) Enlarged views of the tip apex at two different scales, giving the radius of 40 nm. d) Typical normal forces as functions of separations measured during approaching (○) and retracing (◇) process, showing the existence of adhesive force between AFM tip and HOPG. The approach and retrace velocities were set to 200 nm s^−1^. e) Histogram of adhesive force extracted from the 144 normal force curves in an area of 1200 × 1200 nm^2^. The full line is the fit of normal distribution, giving an adhesive force of 17.9 ± 0.9 nN. f) HRTEM image of the AFM tip apex after the presliding. g) Enlarged view of the HRTEM image in the black dashed square marked in (f).

After the superlubricity test, the topography of the tip apex was investigated using field emission scanning electron microscopy (FESEM), as shown in **Figure** **2**a–c. A clear wear was observed on the tip apex, resulting in the tip radius increasing from 7 to 40 nm. The average contact pressure in the superlubricity state is calculated as *P* = *F*_n_/*πr* ^2^, where *F*_n_ is the applied load and *r* is the radius of the contact zone, which can be determined from the Johnson–Kendall–Roberts (JKR) theory^14^

(1) $$r^{3}=\frac{3R\left(F_{n}+F_{a}+2\sqrt{F_{a}^{2}+F_{n}F_{a}}\right)}{4E^{\prime }}$$

where *E*′ is the reduced Young's modulus between silicon and graphite, and *F*_a_ is the adhesive force between the worn tip and graphite, which was measured at different positions as the tip detached from the HOPG (pull‐off force in Figure 2d). The adhesive force mapping in an area of 1200 × 1200 nm^2^, extracted from 144 normal force curves after the presliding shows that the adhesive force is independent of the contact positions (Figure S4, Supporting Information). The statistical results extracted from Figure S4 (Supporting Information) give the adhesive force of *F*_a_ = 17.9 ± 0.9 nN, according to the fit of normal distribution in Figure 2e. Thus, Equation (1) gives the radius of the contact zone as 14.98 nm and so the average contact pressure is 2.13 GPa under a load of 1500 nN. This result indicates that the robust superlubricity of graphite can be achieved even when the average contact pressure exceeds 2 GPa.

In another way, the adhesive force between two surfaces can also be estimated by JKR theory in Equation (2)

(2) $$F_{a}=\frac{3\pi RW_{\mathrm{SG}}}{2}$$

where *R* is the radius of the tip, and *W*_SG_ is the dispersive work of adhesion per unit area of silicon tip in contact with HOPG, which can be expressed as $W_{\mathrm{SG}}=2\sqrt{\gamma_{\mathrm{Si}}^{d}\gamma_{\mathrm{Gra}}^{d}}$, where $\gamma_{\mathrm{Si}}^{d}$ and $\gamma_{\mathrm{Gra}}^{d}$ are the surface energies of the silicon tip and HOPG, respectively. The surface energy of graphite measured in ambient conditions is $\gamma_{\mathrm{gra}}^{d}\approx 54.8\;m\;J\;m^{-2}$,^15^ and so the surface energy of the worn region on the tip can be calculated as $\gamma_{\mathrm{Si}}^{d}\approx 41.2\;m\;J\;m^{-2}$. This is much lower than that of silicon,^16^ indicating that the surface properties of silicon tip were modified after the friction test. Many previous studies have shown that the topmost layers of the graphite can be transferred onto the opposite sliding surface, leading to a significant reduction in surface energy and friction.^6^, ^10^, ^17^ Moreover, the surface energy of graphene is about 46.7 mJ m^−2^ under ambient conditions,^15^ which is very close to the value of $\gamma_{\mathrm{Si}}^{d}$. Therefore, it can be inferred that GNFs are exfoliated from the topmost layer of graphite and transferred onto the worn region of tip after the friction test.

To obtain the further information about the transferred GNFs, the worn surface of the tip was investigated using the cross‐sectional high‐resolution transmission electron microscope (HRTEM), as shown in Figure 2f,g. The radius of the cross‐sectional profile of the worn surface was about 40 nm, which agrees with the FESEM measurement. A transfer film with a thickness of ≈2.2 nm was observed to cover on the worn region of the tip, with some clear features showing that the film is a layered structure with a spacing of 0.37 nm (Figure 2g). This confirms that the topmost layers of the graphite were transferred onto the worn region of the tip, forming GNFs after the sliding process. The spacing of the multiple layers was slightly larger than that of pristine graphite (0.33 nm),^18^ which may be attributed to the mechanical deformation under high contact pressure during the triboexfoliation process.

According to the above results, GNFs are transferred onto the silicon tip, causing the shear plane to shift from silicon/graphite to graphene/graphite because of its lower shear strength. In this case, the GNFs may form the incommensurate sliding with the original graphite substrate because of their similar lattices. Superlow friction would subsequently be achieved because of the extremely weak interaction and easily sliding between the transferred GNFs and graphite in the incommensurate contact.^6^, ^7^, ^10^, ^19^ According to the previous studies on the superlubricity of graphite, the superlow friction would disappear at two certain sliding orientations with an angle difference of 60° under small loads.^6^, ^7^ However, we did not observe this sliding orientation dependency of the superlubricity under the high contact pressure. This is probably because the two lattices of GNFs and graphite are not perfectly rigid, which may result in some mismatches between the two contact lattices under high contact pressures (large deformations).^20^ In this case, the commensurate contact cannot be formed at any sliding orientations, and thus, the superlow friction is independent of the sliding orientation.

**Figure 3:**
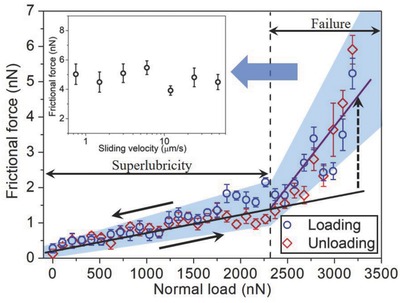
Frictional force as a function of normal load between AFM tip and HOPG, measured during loading and unloading process. The maximal normal load reached 3200 nN, corresponding to an average contact pressure of 2.86 GPa, and the sliding speed was set to 3 µm s^−1^. The black line is the linear fit to the points where the load is in the range of 0–2300 nN, giving a base line of frictional force versus load. The purple line is the linear fit to the points where the load exceeds 2300 nN. The dotted arrow indicates the deviation of the frictional force from the base line. The inset shows the relationship between the frictional force and sliding velocity (in the range of 0.7–50 µm s^−1^) at a constant normal load of 3100 nN.

Since the robust superlubricity of graphite was maintained under such high contact pressure (2.13 GPa), it raises the question of the limiting pressure for the superlubricity of graphite. To answer this question, the frictional forces were measured continuously until the load increased to a very high value (3200 nN), as shown in **Figure** **3**. The frictional force first increased slowly with the load of less than 2300 nN, and then suddenly increased much faster when the load exceeded 2300 nN. The linear fit of the frictional values for loads in the range 0–2300 nN gives a baseline of frictional force versus load. It was observed that the frictional force exhibited a significant deviation from this base line when the load exceeded 2300 nN, implying that there is an extra channel for the frictional energy dissipation at ultrahigh contact pressures. Moreover, this extra frictional energy dissipation was independent of sliding speed (Figure 3, inside). The friction coefficients, based on the linear fit to these points, increased accordingly from *µ* = 0.0004 ± 0.0001 to *µ* = 0.004 ± 0.001 when the load exceeded 2300 nN. It indicates that the limiting applied load (*F*_limit_) for the superlubricity of graphite is about 2300 nN, corresponding to an average contact pressure of 2.52 GPa. When the contact pressure exceeds this critical value, the friction coefficient increases 10 times approximately, signifying the failure of superlubricity.

Because sliding initially occurs between the transferred GNFs and HOPG at loads of less than *F*_limit_, there are two possible factors to cause the extra frictional energy dissipation when the load exceeds *F*_limit_: i) the detachment of the transferred GNFs from the tip and ii) the appearance of wear on the HOPG. To verify the first possibility, the frictional forces were measured continuously with the load reducing from 3200 nN to zero gradually (unloading process). Figure 3 shows that the frictional force decreased rapidly as the load reduced from 3200 to 2300 nN, and then decreased very slowly for load from 2300–0 nN, which is virtually superimposed with that measured during the loading process. This result indicates that the superlubricity can be recovered when the load falls below *F*_limit_ during the unloading process. Even the friction process (loading and unloading) was repeated for more than five times at the same scanning area, the superlubricity can always be recovered once the load falls below *F*_limit_. Moreover, the frictional force had no sudden increase or decrease in the whole scanning area during the unloading process, which implies that the shear plane did not shift between graphene/graphite and silicon/graphite. Therefore, it is inferred that the transferred GNFs were not detached from the tip during the sliding process at ultrahigh contact pressures.

To verify the second possibility, the frictional forces were measured at a high constant load of 3100 nN (contact pressure = 2.82 GPa) continuously over a scanning area of 800 × 800 nm^2^ for 24 circles. The frictional force was maintained at a level of 3.5–5 nN during the whole scanning process (Figure S5a, Supporting Information). The topography of the scanning region was then measured by AFM (Figure S5b, Supporting Information), with the result showing that there was no obvious wear in the scanning region. This indicates that no wear occurs on the HOPG during the sliding process, even under an ultrahigh contact pressure. Therefore, neither of these two factors is the key source of the extra frictional energy dissipation under ultrahigh contact pressures, indicative of the existence of other factors causing the extra frictional energy dissipation.

## 3 Discussions

**Figure 4:**
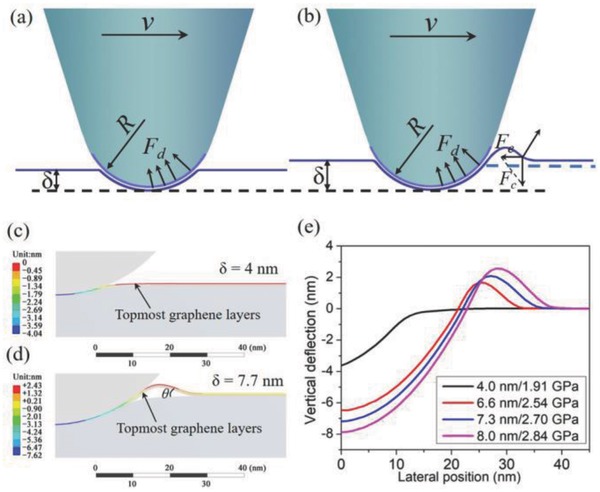
a) Illustration of AFM tip sliding on the graphite under a relative low contact pressure. b) Illustration of AFM tip sliding on the graphite under a very high contact pressure, showing the formation of the pucker ahead of the tip. c,d) FEM simulation results of the tip slides on the HOPG substrate under two different deformations. The topmost graphene layers are not delaminated when c) the deformation is about 4.0 nm, and d) they are delaminated when the deformation reaches 7.7 nm. The angle θ is the inclination angle of the lifted region. e) FEM simulation results of relationship between vertical deflection of topmost graphene layers and lateral positions under four different deformations/pressures.

At the nanoscale, the frictional force (*F*_s_) between the transferred GNFs and graphite can be estimated as *F*_s_ = *τA*, where τ is the shear strength between the transferred GNFs and graphite, and *A* is the contact area (*A* = *πr* ^2^) under an applied load. However, because the deformation of the graphite substrate reaches 6.5 nm in our case when the load reaches *F*_limit_, the influence of the deformation on the frictional force cannot be ignored. It has been demonstrated that the lateral displacement of the deformed graphene beneath the tip can cause the extra frictional energy loss during sliding (even for a deformation moving in the absence of a tip).^21^ Therefore, when the sliding occurs under an ultrahigh contact pressure, the displacement of the largely deformed region requires the tip to provide the additional energy (**Figure** **4**a). Thus, the frictional force between the GNFs and graphite under ultrahigh contact pressure can be estimated by Equation (3)

(3) $$F_{s}=\tau A+F_{d}$$

where *F*_d_ is the additional lateral force produced by the deformation, which could be obtained approximately by Equation (4) for a narrow contact force sweep

(4) $$F_{d}=\frac{1}{2}K\delta^{2}$$

where δ is the deformation of the graphite layer, *K* is the contact stiffness between the tip and graphite. When the normal load (*F*_n_) is much higher than the adhesive force, δ can be described by $\delta ={\left(\frac{9F_{n}{}^{2}}{\mathrm{16}R{E^{\prime }}^{2}}\right)}^{1/3}$, and *A* can be described by $A=\pi {\left(\frac{3RF_{n}}{4E^{\prime }}\right)}^{2/3}$approximately according to the Hertz contact theory. Thus, the relationship between the frictional force and normal load can be obtained according to Equation (3)

(5) $$F_{s}=k_{1}F_{n}^{2/3}+\;k_{2}F_{n}^{4/3}+F_{\mathrm{s0}}$$

where *F*_s0_ is the frictional force at zero load caused by the adhesive force, and *k*_1_, *k*_2_ are the constants determined by τ, *R*, *E*′, and *K*. It indicates that the frictional force increases approximately linearly with respect to the load, which does not obey the atomic friction law ($F_{s}\propto F_{n}^{2/3}$), because the influence of elastic deformation on the frictional force cannot be ignored under very high contact pressures. The frictional force curve in Figure 1c was fitted according to Equation (5) (Figure S6, Supporting Information), which gives the parameters of *k*_1_ = 4 × 10^−6^ and *k*_2_ = 3 × 10^−5^. Thus, the shear strength between GNFs and graphite can be calculated as $\tau =\frac{k_{1}}{\pi }{\left(\frac{4E^{\prime }}{3R}\right)}^{2/3}=0.8$ MPa, which agrees with the measured shear strength between the natural graphite layers reasonably well.^22^ When the load exceeded 2300 nN, the frictional force deviated from the fitted curve, indicative of the existence of some other resistant force in the sliding direction.

As shown in Figure 4b, when the deformation is very large, it is inferred that the topmost graphene layer(s) on the HOPG substrate would be partially delaminated by the AFM tip along the sliding direction because of the weak vdW interaction among graphite's interlayers, which can lead to the formation of a pucker ahead of the tip, similar to a pencil drawing on soft, layered papers. To confirm this inference, we simulated the tip–graphite sliding contact with different deformations/pressures using the finite element method (FEM; Figure S7, Supporting Information). It is observed that there is no pucker formed ahead of the tip when the tip slides on the graphite with a small deformation of 4 nm (Figure 4c), but when the deformation reaches 7.7 nm, a clear delamination of the topmost graphene layers ahead of the tip is observed, which leads to the formation of a lifted region around the tip (Figure 4d). Moreover, both the height and width of the lifted region increase as the deformation/pressure becomes larger, which can reach 2.6 and 19 nm, respectively, when the deformation exceeds 8 nm. Therefore, the additional driving force (*F*_e_) is required to push the lifted region forward, because the delamination requires the tip to provide the additional exfoliation energies during the sliding. In this case, the frictional force under ultrahigh contact pressures (>2.52 GPa) can be described by Equation (6)

(6) $$F_{s}=\tau A+K\delta^{2}/2+F_{e}$$

According to Figure 4b, *F*_e_ can be described by *F*_e_ = *F*_c_ tan θ = *E*_c_(*d* + 2*L*_w_)tan θ, where *F*_c_ is the normal force required to delaminate the topmost graphene layers, θ is the inclination angle of the lifted region (Figure 4d), *E*_c_ is the exfoliation energy of graphite (*E*_c_ = 0.37 J m^−2^),^23^ *d* is the diameter of the contact zone, and *L*_w_ is the width of the lifted region. Because *d* and *L*_w_ both increase as the deformation becomes larger, according to the FEM results, *F*_e_ will increase with increasing load under ultrahigh contact pressures, causing the frictional force versus load curve to deviate from the base line in Figure 3. Considering the case with the maximal contact pressure of 2.86 GPa, the FEM result gives *d* = 38 nm, *L*_w_ = 19 nm, and tanθ = 0.2; thus, we calculated *F*_e_ = 5.62 nN, which is in accordance with the deviation of the frictional force in Figure 3. The remarkable consistency indicates that the required exfoliation energies due to the movement of the pucker ahead of the tip are the main reason why the friction coefficient increases 10 times when the load exceeds 2300 nN.

Our results provide the evidence that the robust superlubricity of graphite sliding against a silicon tip with the transfer of GNFs can be achieved under an ultrahigh contact pressure of up to 2.52 GPa, even when there is a significant deformation of graphite in the contact zone. This indicates that the extremely low shear strength between GNFs and graphite is independent of the pressure and deformation. However, the contact pressure has a threshold, at which the superlubricity state starts to collapse. When the load exceeds this threshold, there exists the additional frictional energy dissipation, which originates from the delamination of the topmost graphene layers on the graphite, caused by the large deformation and sliding. It requires the additional lateral force to push the lifted region forward, and thus leads to the great increase in the frictional force. Although the friction coefficient increased 10 times after the pressure exceeded the threshold, we did not observe any wear on the graphite, which suggests that the transferred GNFs is able to protect the graphite from wear. This finding may be very useful in designing superlubricity systems in the absence of wear under ultrahigh contact pressures.

## 4 Conclusion

In summary, our work has demonstrated that graphite sliding against GNFs at the nanoscale can exhibit robust superlubricity behavior, even under the ultrahigh contact pressure of 2.52 GPa. The friction coefficient can become as low as 0.0003 because of the extremely low shear strength between graphene and graphite in the incommensurate contact. When the pressure exceeds a certain threshold, the friction coefficient increases 10 times, implying the failure of superlubricity, but no wear occurs on the graphite. We attribute the failure to the formation of a pucker ahead of the tip, caused by the delamination of the topmost graphene layers on the graphite, which requires the tip to provide additional exfoliation energies. The robustness of superlubricity and the wear‐less properties of graphite sliding against graphene under ultrahigh contact pressures have important implications for developing novel solid lubricants with superlubricity properties for application to MEMS/NEMS.

## 5 Experimental Section

*Frictional Force Microscopy Tests*: The friction measurements were performed on the Asylum Research MFP‐3D AFM in contact mode, and a silicon tip (Multi75Al‐G, Budgetsensors) with a normal spring constant of 1–5 N m^−1^ was used to measure the frictional force. A HOPG (0.4° mosaic spread) substrate was freshly cleaved using the adhesive tape to give a clean, atomically flat surface. The spring constant of the tip was determined by the frequency method,^24^ and the lateral detector sensitivity of the tip was obtained using a diamagnetic lateral force calibrator.^25^ The frictional forces were obtained after a short presliding process (to stabilize the frictional forces), which was performed over a scanning area of 600 × 600 nm^2^, with a constant applied load of 515 nN, and a scanning velocity of 3 µm s^−1^. The frictional forces were measured as functions of normal loads by driving the HOPG sliding against the tip as the load increased (loading) and then decreased (unloading) at the same contact position. The scanning area at every load was set as 600 × 40 nm^2^, and the scanning velocity was set in the range of 0.5–50 µm s^−1^. The sliding orientations were varied by rotating the HOPG substrate with an angle range of 120° with respect to the tip. The frictional forces were determined from the difference in the lateral force detector signal in one complete friction loop (20 friction loops were averaged for every load) and the lateral detector sensitivity. The tests were performed at a temperature of 25 ± 2 °C and a relative humidity of 30–60%.

*Normal Force and Adhesive Force Measurement*: The normal force curves were obtained as the probe first approached the HOPG substrate and then retracted from the substrate with a velocity of 200 nm s^−1^. The adhesive force was obtained by measuring the pull‐off forces as the probe detached from the HOPG substrate. The adhesive force mapping was obtained by measuring the pull‐off forces at 144 sites (10 000 nm^2^ per site) over an area of 1200 × 1200 nm^2^.

*Surface Characterization*: The topography of the tip before and after the friction test was measured by FESEM (HITACHI SU8220) under a low voltage to protect the surface from damage. The cross‐sectional image of the tip was measured by HRTEM (JEM‐2100F). For the transmission electron microscope (TEM) measurement, the tip was glued onto a copper sheet that was designed for TEM observations (Figure S8, Supporting Information).

*Finite Element Analysis*: Quasistatic FEM was used to simulate the process of AFM tip scanning in contact with a graphite sample. For the sake of simplicity, only a 2D case was considered, in which a rounded cylinder representing the tip with GNFs (*R* = 40 nm) at its end slid against an elastic sheet (approximating the graphene layers) which adhered to a substrate representing the graphite. The elastic sheet and substrate were both 500 nm in length. The tip was assumed to be a rigid body. The elastic sheet and substrate had a Young's modulus of 18 GPa and a Poisson's ratio of 0.2. To prescribe the tip–graphene and graphene–substrate interactions, the cohesive zone model originated from a Lennard–Jones potential (Supporting Information) was used.[[qv: 13b,26]] During the simulation, the elastic sheet and substrate were both fixed at their edges. The tip first moved down to a certain depth to compress the elastic layer and substrate, and then, slid laterally in the specified direction at that constant depth (Figure S7, Supporting Information). This allowed us to observe the variation of the topmost graphene layers ahead of the tip.

## Conflict of Interest

The authors declare no conflict of interest.

## Supporting information

Supplementary
