# Supplementary material for: Superlubricity of Graphite Sliding against Graphene Nanoflake under Ultrahigh Contact Pressure
Source: Adv Sci (Weinh). 2018 Aug 29;5(11):1800810. doi: 10.1002/advs.201800810 (PMC6247022; doi:10.1002/advs.201800810)
Supplement: Supplementary file 1 — Supplementary [file ADVS-5-1800810-s001.pdf]

Copyright WILEY-VCH Verlag GmbH & Co. KGaA, 69469 Weinheim, Germany, 2018.

ADVANCED SCIENCE
Open Access

Supporting Information

for *Adv. Sci.,* DOI: 10.1002/advs.201800810

Superlubricity of Graphite Sliding against Graphene Nanoflake under Ultrahigh Contact Pressure

Jinjin Li, Jianfeng Li, and Jianbin Luo*

Superlubricity of Graphite Sliding against Graphene Nanoflake under Ultrahigh Contact Pressure

Jinjin Li, Jianfeng Li, Jianbin Luo*

State Key Laboratory of Tribology, Tsinghua University, Beijing, 100084, China

1. Normal load and pressure for superlubricity of graphite or graphene in previous studies

Authors	COF	Load/pressure	Friction pairs
Ruan et.al[1]	0.006	42 nN	Slicon nitride tip/ HOPG
Schwarz et al[2]	0.008±0.005(argon atmosphere) 0.005±0.003(ambient conditions)	40 nN	Tip with amorphous carbon/HOPG
Vu et al[3]	close to zero	15 μN/1.67 MPa	Graphite/graphite
Dienwiebel et al[4]	friction force reduced to 15.2 pN at load of 18 nN	18 nN	Tungsten tip/Graphite
Cihan et al[5]	Ultralow friction force	<2.5 nN	Tip and gold islands/graphite
Mate et al[6]	0.012	2 μN	Tungsten tip/HOPG
Kawai et al[7]	friction force < 100 pN	close to zero	Graphene Nanoribbons/gold
Feng et al[8]	close to zero	< 500 pN	GNFs/graphene

2. Original radius of AFM tip apex measured by HRTEM

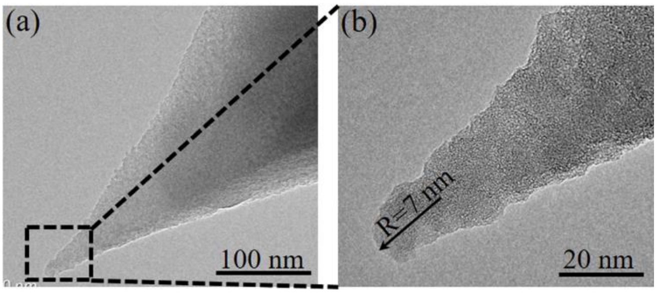

Figure S1 (a) HRTEM image of the AFM tip apex before friction test. (b) Enlarged view of the HRTEM image in the black dashed square marked in a, giving the radius of AFM tip apex of 7 nm.

3. Evolution of frictional force with sliding distance under a constant normal load.

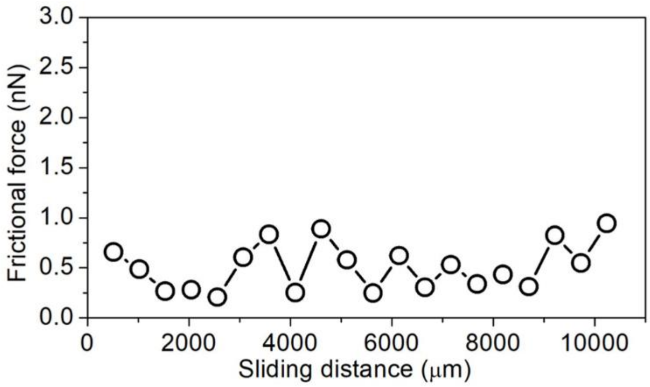

Figure S2 Evolution of frictional force with sliding distance under a normal load of 1030 nN.

4. Relationship between frictional force and sliding orientation

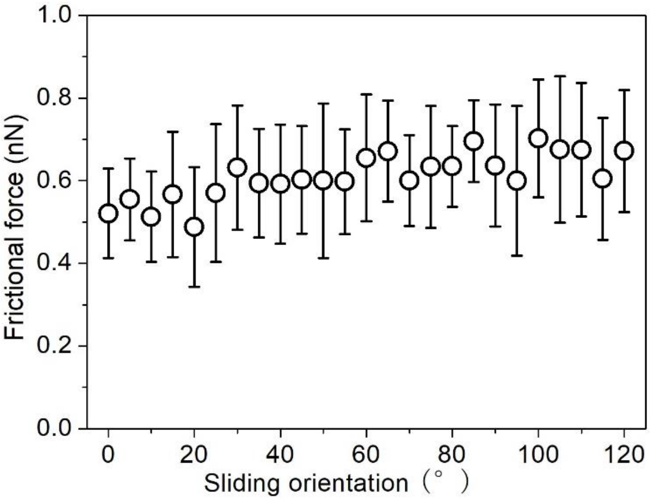

Figure S3 Relationship between frictional force and sliding orientation (varied from 0 - 120°) at a constant normal load of 824 nN and a sliding velocity of 3 μm/s.

5. Adhesive force mapping in an area of 1200 × 1200 nm^2

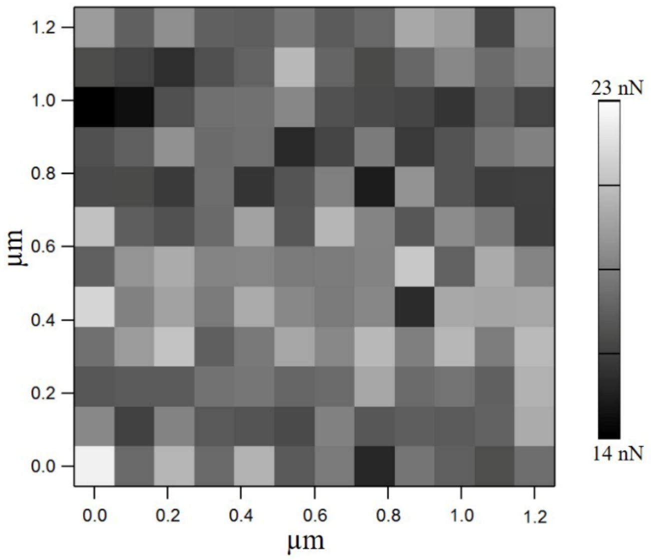

Figure S4 Adhesive force mapping in an area of 1200 × 1200 nm^2, extracted from 144 normal force curves after the presliding, showing that the adhesive force is independent of the contact positions.

6. Evolution of frictional force with scanning circles under an ultrahigh contact pressure and topography after 24 scanning circles

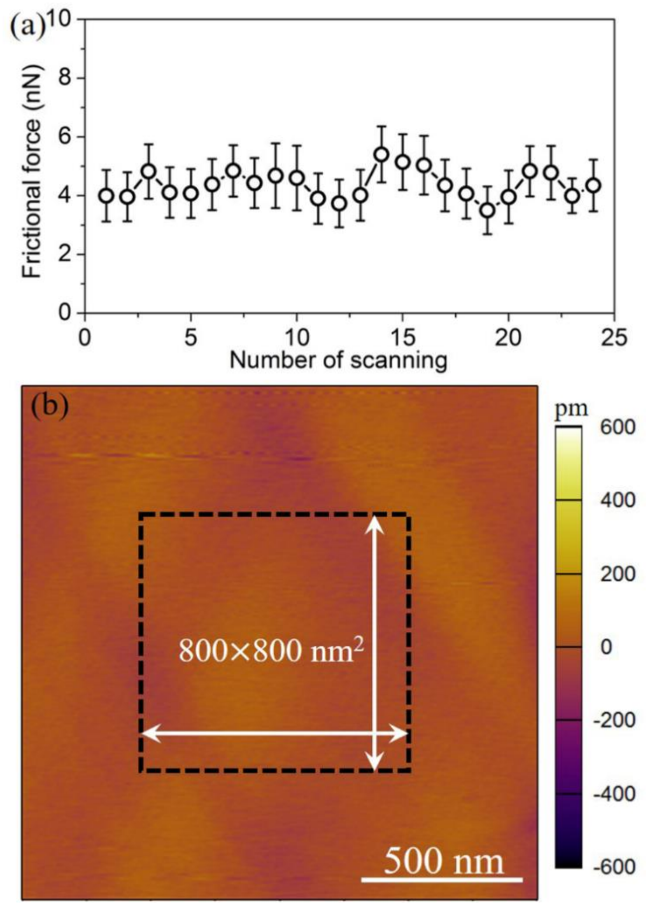

Figure S5 (a) Evolution of frictional force continuously measured over a scanning area of 800×800 nm^2 for 24 circles under an ultrahigh contact pressure of 2.82 GPa. (b) Topography of the scanning region after the 24 scanning circles, showing no obvious wear in the scanning region.

7. Frictional force as a function of normal load (less than 1500 nN) with the fitted line according to Equation (5)

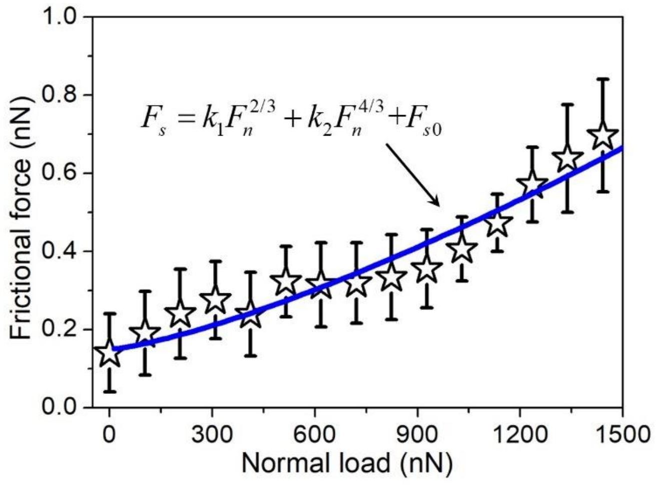

Figure S6 Frictional force as a function of normal load (less than 1500 nN) with the fitted line according to Equation (5), giving fitting parameters of $k_1 = 4\times10^{-6}$ and $k_2 = 3\times10^{-5}$.

8. FEM model for the tip–graphite sliding contact under different deformations

To prescribe tip–graphene and graphene–substrate interactions, we used the cohesive zone model, originated from a Lennard-Jones potential.[9] Specifically speaking, the interaction stress, σ, between the elastic sheet and the substrate can be expressed by

$$\sigma = 3.07\sigma_0\left(\frac{1}{(\delta/\delta_0)^4} - \frac{1}{(\delta/\delta_0)^{10}}\right) \tag{S1}$$

where σ_0 is the adhesion parameter; δ is the inter-surface separation, and δ_0 is the equilibrium separation. In the simulations, the tip–graphene adhesion parameter, σ_0^{t-g}, and the graphene–substrate adhesion parameter, σ_0^{g-g}, were both set to 150 MPa,[10] δ_0 was 0.33 nm for both interfaces here.[11] During the simulation, the elastic sheet and substrate were both fixed at their edges. The tip first moved down to a certain depth to compress the elastic layer and substrate, and then, slid laterally in the specified direction at that constant depth, as shown in Figure S7.

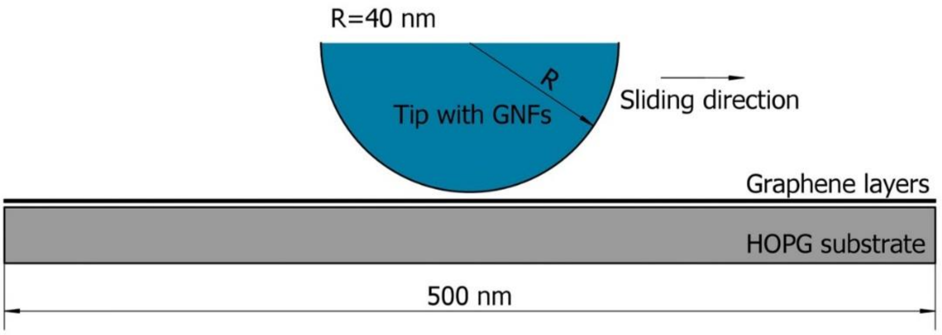

Figure S7 FEM Model of the tip–graphite sliding contact, showing the rigid tip with the radius of 40 nm and the graphene layers with the length of 500 nm adhered to a substrate.

9. Structure of the designed copper sheet to fix the AFM tip for TEM observation

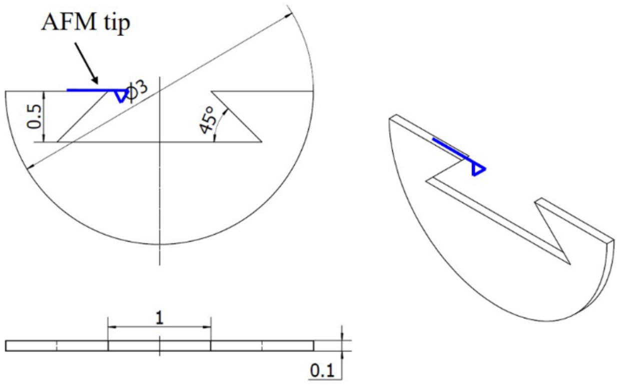

Figure S8 Structure of the designed copper sheet to fix the AFM tip for TEM observation

References

[1] J. A. Ruan, B. Bhushan, *J. Appl. Phys.* **1994,** *76*, 8117.

[2] U. D. Schwarz, O. Zworner, P. Koster, R. Wiesendanger, *Phys. Rev. B* **1997,** *56*, 6987.

[3] C. C. Vu, S. Zhang, M. Urbakh, Q. Li, Q. C. He, Q. Zheng, *Phys. Rev. B* **2016,** *94*, 081405.

[4] M. Dienwiebel, G. S. Verhoeven, N. Pradeep, J. W. Frenken, J. A. Heimberg, H. W. Zandbergen, *Phys. Rev. Lett.* **2004,** *92*, 126101.

[5] E. Cihan, S. Ipek, E. Durgun, M. Z. Baykara, *Nat. Commun.* **2016,** *7*, 12055.

[6] C. M. Mate, G. M. McClelland, R. Erlandsson, S. Chiang, *Phys. Rev. Lett.* **1987,** *59*, 1942.

[7] S. Kawai, A. Benassi, E. Gnecco, H. Soede, R. Pawlak, X. Feng, K. Muellen, D. Passerone, C. A. Pignedoli, P. Ruffieux, R. Fasel, E. Meyer, *Science* **2016,** *351*, 957.

[8] X. Feng, S. Kwon, J. Y. Park, M. Salmeron, *ACS Nano* **2013,** *7*, 1718.

[9] L. Y. Jiang, Y. Huang, H. Jiang, G. Ravichandran, H. Gao, K. C. Hwang, B. Liu, *J. Mech. Phys. Solids* **2006,** *54*, 2436.

[10] Z. Deng, A. Smolyanitsky, Q. Li, X. Q. Feng, R. J. Cannara, *Nat. Mater.* **2012,** *11*, 1032.

[11] G. E. Bacon, *Acta Crystallogr.* **1951,** *4*, 558.
